# Effect of Implementation of the Lockdown on the Number of COVID-19 Deaths in Four European Countries

**DOI:** 10.1017/dmp.2020.433

**Published:** 2020-11-04

**Authors:** Raffaele Palladino, Jordy Bollon, Luca Ragazzoni, Francesco Barone-Adesi

**Affiliations:** 1 Department of Public Health, University “Federico II” of Naples, Italy; 2 Department of Primary Care and Public Health, Imperial College, London, UK; 3 CIRMIS – Interdepartmental Center for Research in Healthcare Management and Innovation in Healthcare, University “Federico II” of Naples, Italy; 4 Department of Translational Medicine, Università del Piemonte Orientale, Novara, Italy; 5 CRIMEDIM – Research Center in Emergency and Disaster Medicine, Università del Piemonte Orientale, Novara, Italy

**Keywords:** COVID-19, lockdown, evaluation, late implementation, healthcare, health

The coronavirus disease (COVID-19) epidemic has rapidly evolved into a global health emergency.^1^ However, there has been a substantial heterogeneity in timing and magnitude of the public health response to the pandemic among different countries.^1–4^ A key factor in explaining why China was successful in curbing the epidemic is that the government implemented containment measures in the Hubei Province in the very early phase of the epidemic.^2^ On the contrary, Europe has been slower in responding to the emergency. In France, Italy, Spain, and the UK, the 4 European countries that have been impacted the most by the COVID-19 emergency, lockdown was enforced 13 to 16 days after Hubei’s, when normalizing for the time when the outbreak hit 50 cases in all countries.^2^ This prompts the question of how many COVID-19 deaths could have been avoided during the early phase of the pandemic if containment measures in European countries had aligned with China’s lockdown.

We modeled the daily number of COVID-19 deaths in France, Italy, Spain, and the UK from January 23 to August 15. Data were downloaded from the COVID-19 Data Repository.^1^ The time-series included a small amount of days in which the number of deaths was negative. We considered these figures as missing values and replaced them, interpolating the number of deaths recorded in the previous and in the following day. We estimated the effect of the national lockdown implementing an Interrupted Time Series analysis.^5^ Specifically, for each country, we modeled the time-series of daily deaths (Y_t_) using the following quasi-Poisson regression model:

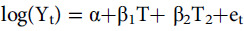

where T is the time elapsed since the start of the observation period (January 23); T_2_ is the time elapsed since the implementation of the lockdown (set to 0 before the lockdown); Y_t_ is the number of new deaths at time T; α is the intercept of the model; β_1_ represents the trend of new cases before the lockdown; β_2_ is the slope change following the lockdown; and e_t_ is the error term of the model.

To take into account the COVID-19 incubation period and the mean time between the symptoms onset and death, we assumed an 18-day lag between the national implementation of the lockdown and the start of its effects.^6^ Then, we created 4 separate counterfactual scenarios by predicting the daily number of deaths that would have been observed in the 4 countries if the lockdown had been implemented at the same time as Hubei’s (3 days after the outbreak hit 50 cases). This timing would correspond to the following dates: February 25 for Italy, March 2 for France, March 3 for Spain, and March 6 for the UK. Finally, we estimated the relative change in the number of total deaths in the counterfactual scenario, compared to the observed one. All the analyses were performed using R software (R Foundation for Statistical Computing, Vienna, Austria).

Figures 1 and 2 display the daily and total number of deaths in the 4 countries. As of August 15, there were 31 174, 35 449, 30 731, and 41 361 deaths in France, Italy, Spain, and the UK, respectively. If an early lockdown had been implemented, the death toll would have been 2461 (95% CI: 1440 to 4272), 6769 (95% CI: 5652 to 8135), 6792 (95% CI: 4154 to 11 525), and 4071 (95% CI: 3281 to 5067), corresponding to a 92% (95% CI: 86% to 95%), 81% (95% CI: 77% to 84%), 78% (95% CI: 62% to 86%), and 90% (95% CI: 88% to 92%) relative reduction, as compared with observed data.

**Figure 1.**
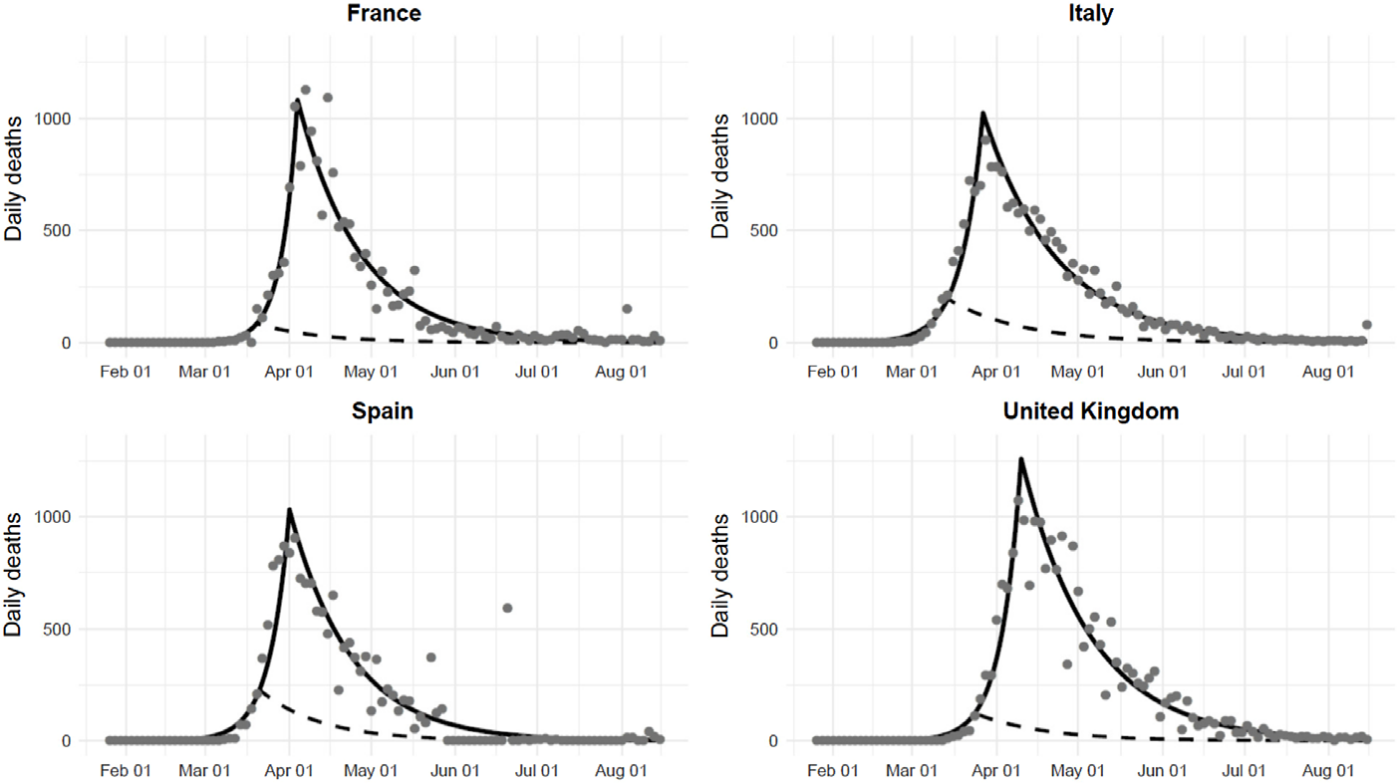
Observed (gray points), fitted (black line), and predicted (dashed line) number of new deaths in France, Italy, Spain, and the UK, assuming an early implementation of the national lockdown (3 days after the outbreak hit 50 cases in each country). Observed points were averaged over 3 days.

**Figure 2.**
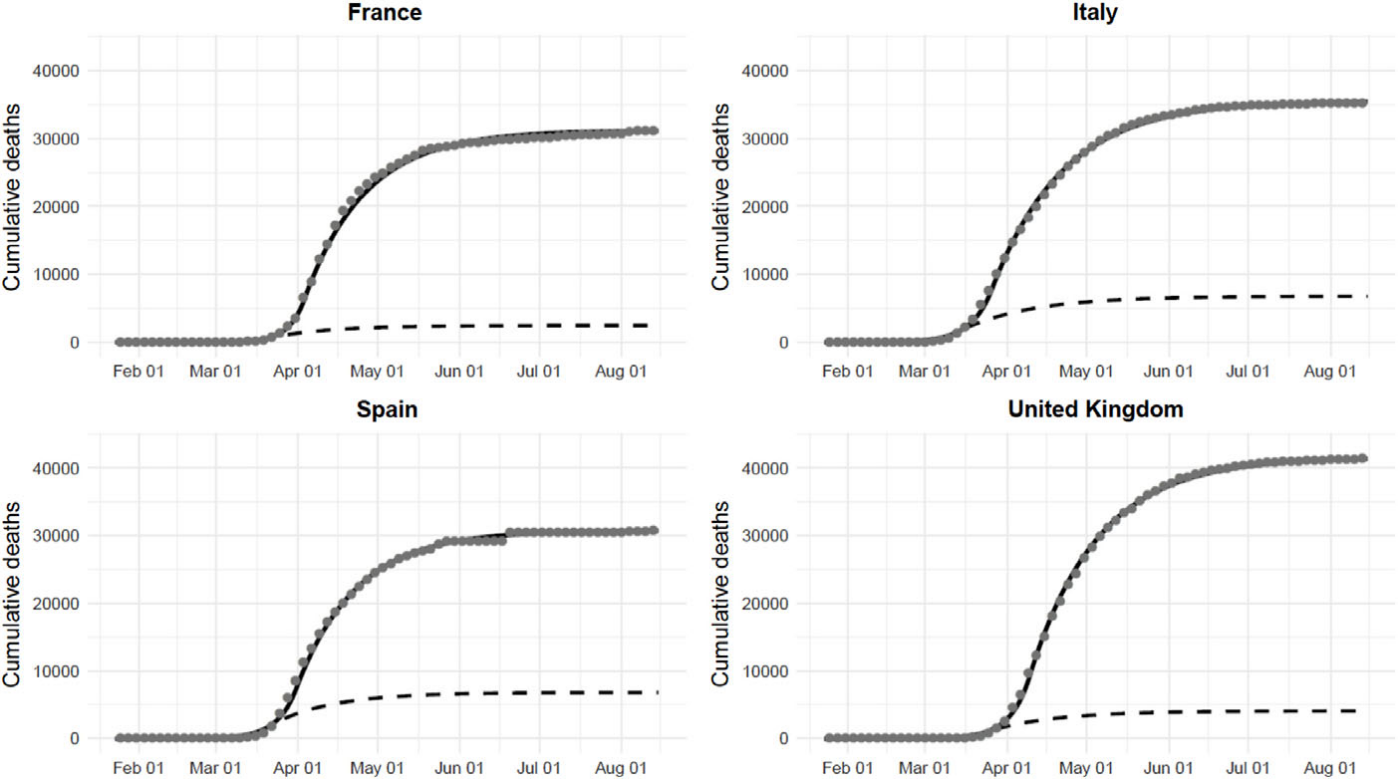
Observed (gray points), fitted (black line), and predicted (dashed line) total number of deaths in France, Italy, Spain, and the UK, assuming an early implementation of the national lockdown.

We found that a more rapid and homogeneous response would have avoided a substantial number of deaths. Our results underline the need of strengthening public health emergency preparedness at national and global levels. Currently, no health care system can sustain an uncontrolled epidemic of COVID-19 or similar disease. Community containment measures are still the most important interventions to minimize the health impact of a possible second wave of infections, avoiding the unnecessary loss of lives.

## Data Availability

The data underlying this article are publicly available from the COVID-19 Data Repository at: https://github.com/CSSEGISandData/COVID-19/tree/master/csse_covid_19_data/csse_covid_19_time_series.
